# Effect of Nanopore Length on the Translocation Process of a Biopolymer: Numerical Study

**DOI:** 10.3390/ma6093989

**Published:** 2013-09-11

**Authors:** Suresh Alapati, Woo Seong Che, Yong Kweon Suh

**Affiliations:** 1 Department of Mechatronics Engineering, Kyungsung University, 309 Suyeong-ro, Nam-gu, Busan 608-736, Korea; E-Mails: sureshalapatimech@gmail.com (S.A.), wsche@ks.ac.kr (W.S.C.); 2 Department of Mechanical Engineering, Dong-A University, 840 Hadan-dong, Saha-gu, Busan 604-714, Korea

**Keywords:** translocation motion, bio-polymer, nanopore length, lattice Boltzmann method

## Abstract

In this study, we simulate the electrophoretic motion of a bio-polymer through a synthetic nanopore in the presence of an external bias voltage by considering the hydrodynamic interactions between the polymer and the fluid explicitly. The motion of the polymer is simulated by 3D Langevin dynamics technique by modeling the polymer as a worm-like-chain, while the hydrodynamic interactions are incorporated by the lattice Boltzmann equation. We report the simulation results for three different lengths of the nanopore. The translocation time increases with the pore length even though the electrophoretic force on the polymer is the same irrespective of the pore length. This is attributed to the fact that the translocation velocity of each bead inside the nanopore decreases with the pore length due to the increased fluid resistance force caused by the increase in the straightened portion of the polymer. We confirmed this using a theoretical formula.

## 1. Introduction

Solid-state or synthetic nanopores have emerged as next generation bio-sensors for the electrical detection, analysis, and manipulation of single biomolecules such as DNA, RNA, and proteins. These pores are mainly used in the nanopore sequencing method, which has great potential to become a high throughput cost-effective technique for DNA/RNA sequencing. In this technique, the sequence of bases along a DNA strand can be detected by translocating the DNA molecule through a nanopore using an external electric field [1]. By allowing direct reading of the DNA sequence, this technique is many times faster than presently used methods as there is no need for PCR amplification or fluorescent labeling [2]. Use of this technique can reduce the costs incurred by genome analysis significantly. Understanding the dynamics of the translocation process is necessary for developing new and improved methods for DNA sequencing. Thus, many researchers are paying considerable attention to explore the mechanism of polymer translocation.

Kasianowicz *et al.* [1] conducted the translocation experiments for the first time. They used the *α*-hemolysin (*α*-HL) nanopore of diameter 2.6 nm to translocate a single stranded DNA using an externally applied electric field. The translocation velocity, *v_t_*, was found to be around *v_t_* = 0.55 mm/s. Several drawbacks (limited lifespan, lack of robustness, difficulty of adjusting pore size and shape) of using biological (*α*-HL) nanopores have driven later many researchers to use synthetic ones. Synthetic nanopores with variable sizes and shapes can be made from silicon oxide (SiO2) or silicon nitrate (Si3N4) membranes using nanofabrication technology, and are very robust compared to their biological counterparts. Li *et al.* [3] used a synthetic nanopore with a diameter of 10 nm made from Si3N4 membrane in their translocation experiments. They studied the translocation process of a double-stranded DNA (dsDNA) and found that *v_t_* is in the order of 10mm/s for dsDNA with lengths of 3 and 10 kilo base pairs (kbp). This value of *v_t_* is about 20 times higher than the one obtained in [1]. The reason for this was explained by Storm *et al.* [4], who conducted translocation experiments using the SiO_2_ nanopore with a diameter of 10 nm by varying the length of dsDNA from 6.6 to 97 kbp. They mentioned that the effective friction between the polymer and pore molecules is very high in the *α*-HL pore compared to that in the SiO_2_/Si_3_N_4_ nanopore because the diameter of *α*-HL is in the same order of magnitude as that of the dsDNA (2 nm). Due to this effective friction, *v_t_* in the *α*-HL pore is very low compared to that in the synthetic nanopore. Storm *et al.* also found that there is a non-linear (power-law) relationship between translocation time and contour length of the polymer, *τ_t_* ~ $L_{c}^{\alpha }$ with the exponent *α* = 1.26, which means that the translocation velocity depends on the polymer length like *v_t_* ~ *L_c_*^−0.26^.

Many researchers conducted theoretical and numerical studies on the polymer translocation process to match the *α* value obtained in [4]. Muthukumar [5] used a theoretical study to find the *α* value of the translocation process of a biopolymer, which was driven by a chemical potential gradient. He obtained *α* = 2 and *α* = 1 in the weak and strong driving force regimes, respectively. Vocks *et al.* [6] conducted 3D numerical simulations without considering the hydrodynamic interactions (HI) and obtained *α* = 1.37. Here, “without HI” means that the fluid is quiescent, but a frictional force exists between the polymer and the fluid. Forrey *et al.* [7] used 3D Langevin dynamics (LD) technique to simulate the translocation process of dsDNA through synthetic nanopores and obtained *α* = 1. Edmonds *et al.* [8] investigated the effect of the chain length, pore geometry (diameter and length), and the applied bias voltage ∆*φ* on the scaling exponent of the translocation process by 3D LD technique. They considered an atomistically detailed structure for the nanopore and used the realistic parameters in their study. They conclude that when the pore length is much smaller than the polymer length, *α* value depends on both the polymer length and applied bias voltage. However, for the case of long pores they found that the scaling law is *τ_t_* ~ ∆*φ*^−1^ for all the voltage values and polymer lengths. Most of the studies discussed so far did not consider the effect of HI, *i.e.*, the fluid flow effect. There are many mesoscopic methods such as the lattice Boltzmann equation (LBE) [9,10], Brownian dynamics [11], dissipative particle dynamics [12], and multi-particle collision dynamics [13] are available to solve the fluid flow in the translocation process. Fyta *et al.* [14,15] have simulated the polymer translocation process by solving the fluid flow using LBE, and they could match their value of *α* = 1.29 with the experimentally observed one *α* = 1.26 [4]. Fyta *et al.* [16,17,18] have also conducted several numerical studies to know the effect of various parameters such as pore size [16,17], and external electric field (driving force) [18] on the translocation process. They observed that biopolymers undertake multi-folded configurations when they are passing through wide nanopores [16,17]. Alapati *et al.* [19,20] conducted numerical studies on the translocation process by considering the HI explicitly using LBE. They could match the value *α* = 1.24 as well as the results of the translocation time and velocity with the corresponding experimental values [4]. They also found that the reason for the variation of translocation velocity relative to the polymer length is due to the variation of the fluid drag force acting on the polymer on the *cis* side [20].

Identifying the sequence of the bases of DNA travelling at 10 mm/s (translocation velocity observed in [3,4]) requires an electronic sensing system with an extremely high bandwidth [21]. High-bandwidth signals generally have very high electronic noise, which limits the electrical detection of the bases of a biopolymer using the nanopore sequencing technique. So, slowing down the velocity of molecules passing through the nanopore is an essential step in the detection and analysis of the single molecules. There are many factors affecting the translocation velocity inside the nanopore, including solvent viscosity, the salt concentration, the applied voltage across the nanopore [21], and the pore size and shape [22]. Fologea *et al.* [21] studied the effect of fluid temperature, KCl concentration, viscosity, and the electrical bias voltage on the translocation speed of a DNA molecule inside a Si_3_N_4_ pore. By increasing the fluid viscosity and decreasing the bias voltage and the temperature of the fluid, they could reduce the translocation speed by an order of magnitude compared to the previous studies. Most of the studies to date used short nanopores around 20 nm in length. Liu *et al.* [22] used a very long nanopore (100 nm long) with a conical shape fabricated with the Si_3_N_4_ membrane and found that the translocation speed can also be reduced by increasing the length of the pore without changing the other parameters. They found that the translocation speed in a nanopore with 100 nm long is five times slower than that in a 20 nm long nanopore.

In this study, we simulate the translocation process of a bio-polymer through nanopores driven by an applied bias voltage. We use LBE to include the effect of HI between the polymer and the fluid. This work is the extension of our earlier work [19,20]. The main aim of this study is to know the effect of nanopore length on the polymer’s translocation speed inside the pore and to find the reason for the decrease of the translocation velocity within the elongated nanopore. The remainder of this paper is arranged as follows: Section 2 gives a brief explanation about the simulation scheme employed in the present study. The numerical results obtained from the simulation of the polymer translocation process through nanopores of different lengths are presented in Section 3. The concluding remarks of the present study are given in Section 4.

## 2. Method of Numerical Simulation 

### 2.1. Simulation Set-Up 

**Figure 1:**
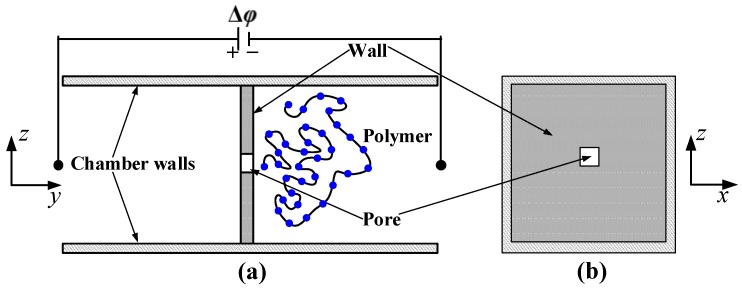
Sectional view illustrating the simulation set-up used in the present work: (**a**) *yz*-plane and (**b**) *xz*-plane.

Figure 1 shows the numerical set-up employed in the present work. All the simulations are carried out over the domain inside a 3D channel. A wall, which divides the channel into two separate chambers in the *y-*direction, is placed at the middle of the channel. A square nanopore through which the polymer translocates is created in the center of the wall. In Figure 1, ∆*φ* represents the applied potential difference which drives the polymer from the *cis* side to the *trans* side of the chamber. A brief explanation for the numerical method is given below.

### 2.2. Numerical Method

**Figure 2:**
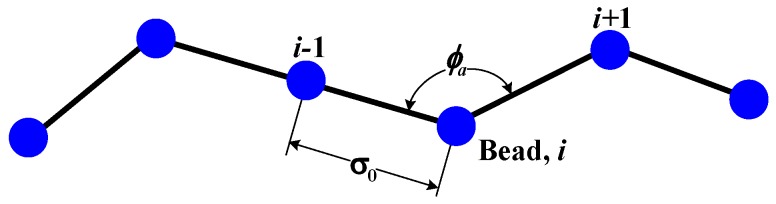
Polymer chain model used in this study for discretization of dsDNA molecule.

In this work, we represent a dsDNA molecule on a coarse-grained level by using a bead model with a worm-like-chain configuration as shown in Figure 2. The contour length of the polymer *L*_c_ is discretized into *N* number of beads connected by *N* − 1 elastic segments of equal length. The position **r***_i_* and the velocity **v***_i_* of each bead *i* are computed by the generalized Langevin dynamics equation for *i* ranging from 1 to *N* as follows:

(1a) $$\frac{\text{d}r_{i}}{\text{d}t}=v_{i}$$

(1b) $$m_{i}\frac{\text{d}v_{i}}{\text{d}t}=F_{\text{LJ},i}+F_{\text{bond},i}+F_{\text{bend},i}+F_{\text{drag},i}+F_{\text{ran},i}+F_{\text{elec},i}$$

Where *m_i_* is the mass of the bead *i*, *t* is the time, **F**_LJ,*i*_ is the force due to total Lennard-Jones potential, **F**_bond,*i*_ is the bond-stretching force, **F**_bend,*i*_ is the bond-bending force, **F**_drag,*i*_ is the drag force, **F**_ran,*i*_ is the random force, and **F**_elec,*i*_ is the electrophoretic force acting on each bead *i*. The formulas for **F**_LJ,*i*_, **F**_bond,*i*_, **F**_bend,*i*_, and **F**_ran,*i*_ are provided in the supplementary information file [Equations (s1)–(s4)].

The viscous drag force due to the relative motion of the polymer bead in the surrounding fluid is calculated by

(2) $$F_{\text{drag},\;i}=-\Gamma_{\text{bare}}\left(v_{i}-u_{\;i}\right)$$

where Γ_bare_ is the input/bare friction coefficient of each bead and **u***_i_* is the fluid velocity at the bead location. In this work, we used LBM as a fluid-flow solver. Since LBM is a grid-based solver, we need to interpolate **u***_i_* from the values at the surrounding grid points. For this purpose, we use a four-point interpolation function, which is given by [10]

(3) $$w(r)=\left\{ \begin{array}{cc} \frac{1}{8}\left(3-2\left|r\right|+\sqrt{1+4\left|r\right|-4r^{2}}\right) & 0\leq \left|r\right|\leq 1\\ \frac{1}{8}\left(5-2\left|r\right|-\sqrt{-7+12\left|r\right|-4r^{2}}\right) & 1\leq \left|r\right|\leq 2\\ 0 & 2\leq \left|r\right| \end{array}\right.$$

where *r* is the distance from the bead position to a surrounding grid point. Note that Γ_bare_ in Equation (3) is different from the actual friction coefficient Γ (see Reference [19] for more details) and when there is no HI effect, we just set **u***_i_* = 0 and Γ_bare_ = Γ in Equation (3).

**Figure 3:**
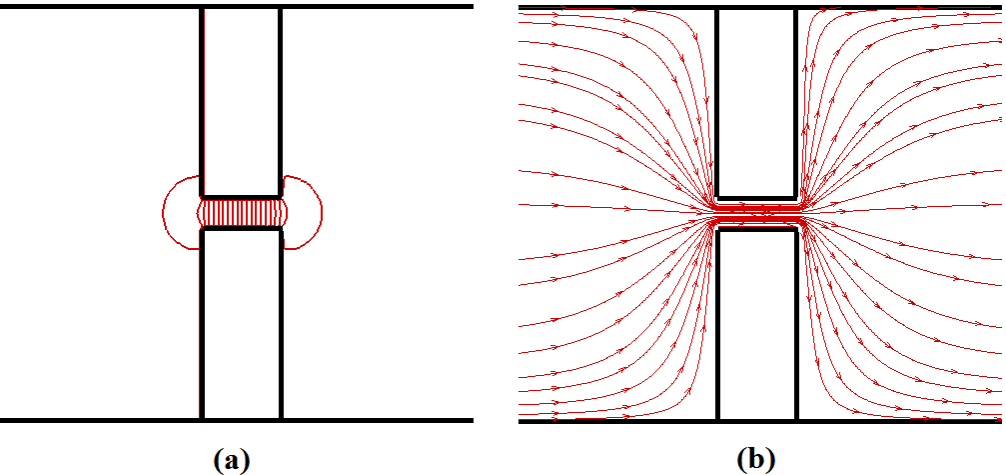
Solution of the Laplace equation for the potential around a 100 nm long nanopore: (**a**) electric potential and (**b**) electric field at the central *yz*-plane.

The electrophoretic force due to an applied potential difference is calculated by

(4) $$F_{\text{elec},\;i}=q_{i}\;E$$

where *q_i_* is the net charge on each bead and ***E*** is the electric field . We use a simple formula for calculating *q_i_*: *q_i_* = *Q_eff_σ*_0_. Here, *Q_eff_* represents the effective charge per unit length of dsDNA [19]. The distribution of the potential difference inside the channel is obtained by solving the Laplace equation ∇^2^*φ* = 0 by using a finite difference method. For boundary conditions, we applied a total potential drop ∆*φ* across the open boundaries (in the *y*-direction) and **n** ∇ *φ* = 0 at the walls; where **n** is the unit vector normal to the walls. The electric field is then calculated from **E** = − ∇ *φ*. ***E*** values at the bead positions are interpolated from the values of the surrounding grid points. Figure 3 shows the solution of the electric potential and electric field for a 100 nm long nanopore. Many researchers [23,24,25] reported that when a nanopore fabricated from a thin insulating membrane (such as SiO_2_/Si_3_N_4_) is immersed in an electrolyte solution and a potential difference is applied across the membrane, most of the voltage drop occurs inside the pore. In other words, the electric field and the resulting electrophoretic driving force are negligible outside the nanopore. Figure 3 also confirms this.

We integrate the LD equations [Equations (1a) and (1b)] for the polymer motion numerically by using the stochastic position Verlet (SPV) algorithm [26] with time step *dt*. Periodic boundary conditions are applied in the *y*-direction, and the interactions with the wall molecules are given by the Lennard-Jones potential. Initially, the bead positions of the polymer are located randomly on the *cis* side of the simulation domain using a self-avoiding random walk algorithm. The position of the first bead is fixed at the entrance of the pore. Then, the polymer motion is simulated without the HI effect until it reaches the equilibrium condition, which corresponds to the condition of the polymer in thermal equilibrium with the fluid. The equilibrium position and the velocity of each bead of the polymer computed in this way are considered as the initial conditions for the subsequent translocation process.

As already mentioned, we use LBE for solving the fluid flow. One can refer to [19,20] for the details of lattice Boltzmann (LB) modeling of fluid motion, coupling the fluid and polymer motion, as well as for the simulation procedure. For LBE also, periodic boundary conditions are applied in the *y*-direction, and the mid-plane bounce-back scheme is employed at the solid walls. The fluid velocity and density at the start of the simulations are taken to be zero and one (in LB units), respectively.

We simulate the translocation process with different chain lengths, *i.e.*, *N* = 51, 102, 223, 320, and 391, which correspond respectively to 1500, 3000, 6557, 9416, and 11500 bp of dsDNA. The simulation domain size is taken as 40 × 80 × 40 (in lattice units) in the *x*, *y*, and *z* directions, respectively, for the polymers with *N* ≤ 200, whereas we have chosen the domain size equal to 50 × 100 × 50 for *N* > 200. We set the size of the nanopore at *σ* = 3*σ*_0_. Due to the repulsive Lennard-Jones interactions between the polymer beads and the pore molecules, the effective hole size is equal to *σ*_0_, rather than 3*σ*_0_. The values for the simulation parameters and the reference quantities used for non-dimensionalization of LD equations [Equations (1a) and (1b)] are given in Table 1 of the supplementary information.

## 3. Simulation Results

In this section, we discuss the simulation results obtained for the translocation of dsDNA through a nanopore with the configuration shown in Figure 1. The translocation process of the polymer is simulated with and without HI by considering different nanopore lengths *L_p_* = 20, 60, and 100 nm. The translocation time *τ_t_* of the polymer chain is defined as the interval between the time when the first bead of the polymer begins to enter the nanopore at *cis* side and the time when the last bead of the polymer leaves the nanopore at *trans* side.

**Figure 4:**
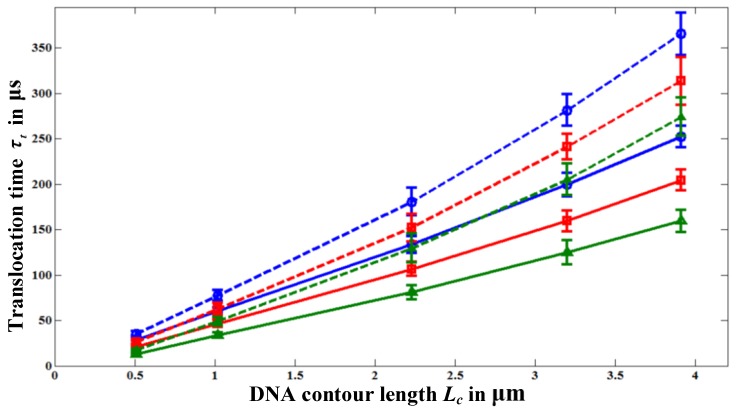
Variation of the translocation time *τ_t_* with polymer length, *L_c_*, for different nanopore lengths; *L_p_* = 100 nm (○), *L_p_* = 60 nm (□), and for *L_p_* = 20 nm (∆).

Figure 4 shows the variation of *τ_t_* with polymer length *L_c_* for various nanopore lengths. Each data point in Figure 4 is the ensemble average over 30–40 translocation events and each event is simulated with a different initial random configuration to reduce statistical uncertainty. For all pore length cases, we started the simulations with the same initial random configurations. It should be noted that the simulations with HI are computationally very time intensive. For example, simulating a single translocation event of a polymer with *N* = 391 beads (11500 bp) through a 100 nm long nanopore requires the computational time of four days on a 2.4 GHz Intel® core 2 processor. So, we simulate 30–40 translocation events to create the ensemble average. Nevertheless, from the error-bars in Figure 4, we can see that our results (particularly for the case with HI) are statistically reasonable. For the nanopore of *L_p_* = 20, we sometimes observed folded (hair-pin) type translocation events as reported in the experiments [4] due to the high electric field intensity |**E**| inside the pore compared to that in the longer pores, *L_p_* = 60 and 100 nm. In taking the ensemble average, we consider only the unfolded translocation events. We also find that the probability for the first bead to enter the pore decreases with the pore length as |**E**| decreases with *L_p_*.

The dashed lines in Figure 4 represent *τ_t_* values when HI effect is neglected. Without HI, *τ_t_* is always greater than the one obtained with HI. This is because the fluid drag force **F**_drag_ acting on the polymer in the case without HI is always larger than the one with HI. The reason for this can be further explained as follows: when HI are neglected, the total friction coefficient of the entire polymer Γ_tot_ is equal to the sum of the individual bead friction coefficient Γ. On the other hand, when the HI effect is considered, Γ_tot_ is less than the sum of the Γ’s of all the beads because of the fluid flow effect. From Figure 4, we can see that *τ_t_* increases with the pore length *L_p_* for the same polymer length *L_c_* even though the polymer receives almost the same amount of electrophoretic driving force, **F**_elec_, irrespective of the pore length. The total driving force acting on the polymer beads inside the nanopore can be approximated as **F**_elec_ = *n_p_q_i_***E**, as the electric field is negligible outside the nanopore (see Figure 3); here, *n_p_* is the number of beads inside the pore. For the same applied potential difference value, **F**_elec_ is independent of the pore length as *n_p_* is proportional to *L_p_* whereas **E** is proportional to 1/*L_p_*. So, the translocation time should depend upon the applied potential difference across the nanopore rather than on the external electric field value. However, in Figure 4, the translocation time increases with the pore length even though the applied voltage is kept the same. To discover the reason for the increase of the translocation time (or the decrease of the translocation velocity) with *L_p_*, one needs to understand the mechanism of the translocation process, which is explained in the following.

**Figure 5:**
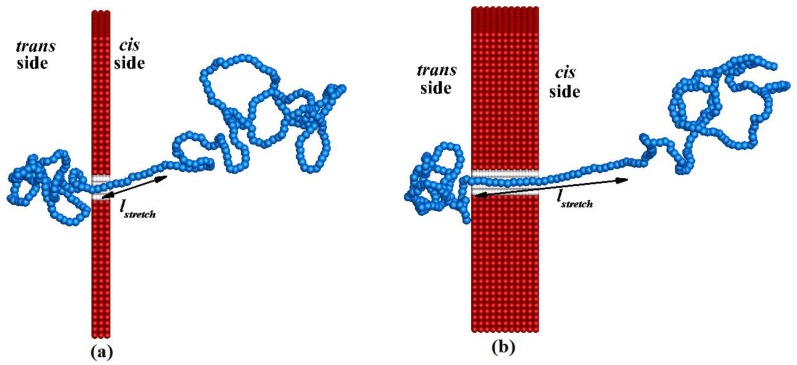
An instantaneous configuration of the polymer of length *N* = 320 while it is translocating from the *cis* side to the *trans* side for pore lengths: (**a**) *L_p_* = 20 nm and (**b**) *L_p_* = 100 nm.

Since the electrophoretic driving force **F**_elec_ mainly acts on the portion of the polymer that resides inside the pore (as the electric field is negligible outside the pore), the entire polymer blob on the *cis* side does not move towards the nanopore at the same time. Instead, only the part of the polymer coil (fold) that is closest to the nanopore feels the effect of **F**_elec_ and has a tendency to move towards the pore. So, **F**_elec_ tends to stretch the portion of the polymer coil closest to the pore. The straightening of all the folds of the initial random configuration on the *cis* side continues successively until the entire translocation process finishes [27]. Note that at any instant of time, only the beads in the straightened portion of the polymer located close to the pore are in forced motion whereas the remaining beads are almost immobile (as they receive only Brownian motion). Figure 5 illustrates the straightened configuration of the polymer of length *N* = 320 while it is translocating from the *cis* side to the *trans* side for pore lengths *L_p_* = 20 nm and *L_p_* = 100 nm. The configurations are snapped when the 120th bead of the polymer is at the end of the corresponding nanopore. In Figure 5, we can see that the length of the straightened (stretched) portion *l*_stretch_ of the polymer closer to nanopore on the *cis* side is greater for *L_p_* = 100 nm than for *L_p_* = 20 nm. The translocation velocity of each bead, *v_t_*_,*i*_, inside the nanopore mainly depends on *l*_stretch_. The stretched portion of the polymer outside the pore and that inside the pore should show slower motion if *l*_stretch_ is larger because the fluid drag force is increased when *l*_stretch_ is increased.

We confirmed this by using a theoretical formula,

(5) $$v_{t,\;i}=\frac{L_{p}}{t_{i}}$$

where *t_i_* is the residence time of bead *i*, which is defined as the time spent by each bead inside the nanopore. The equation for *t_i_* is [20]

(6) $$t_{i}=K\sum_{k=i+1}^{N}\exp\left(-\sum_{j=i+1}^{k}{\left|\theta_{j}\right|}^{\beta }\right)$$

where *θ_j_* is the bent angle of a bead *j*, and *K* and *β* are constants. The above equation was derived by considering the geometrical shape of the polymer on the *cis* side. For a detailed derivation one can refer to [20]. The value of *t_i_* in Equation (6) depends on *l*_stretch_; if *l*_stretch_ is increased *t_i_* is also increased and vice versa. The value of *β* in Equation (6) is obtained from trial-and-error method and we find that for *β* = 3.5, the trend of *v_t_*_,*i*_ distribution calculated from Equation (6) is similar to that obtained from the numerical solution of LD equations. The value of *K* is calculated from the curve fitting (least-squares fit) of data given from Equation (6) and the simulated data.

**Figure 6:**
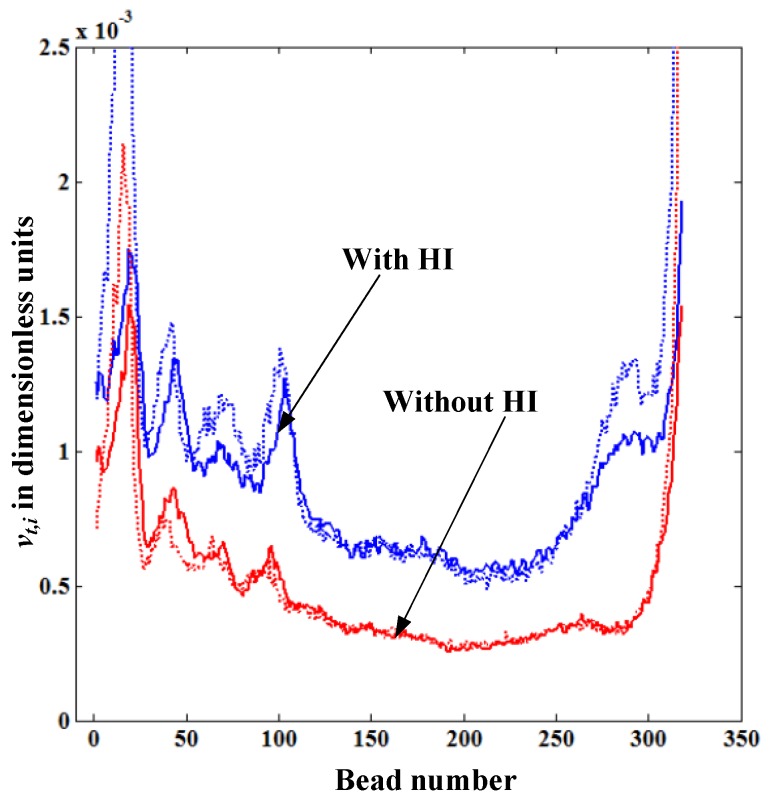
Variation of the translocation velocity of each bead *v_t_*_,*i*_ inside the nanopore for the cases with and without HI for the polymer length *N* = 320. The dotted lines represent the values *v_t_*_,*i*_ calculated from Equation (5).

Figure 6 shows the comparison of the translocation velocity distribution of each bead *v_t_*_,*i*_ given from the solution of LD equations and that obtained from the theoretical formula Equation (5), both with and without HI. In both cases, the simulation begins with the same initial random configuration. The dotted lines in Figure 6 represent *v_t_*_,*i*_ calculated from Equation (5), which is in good agreement with the simulation data for both cases with and without HI. This confirms our physical intuition that the translocation velocity of each bead inside the nanopore mainly depends on the *l*_stretch_ near the pore. In Figure 6, the translocation velocity with HI is always higher than that without HI as the fluid resistance (drag force) on the straightened portion of the polymer without HI is always larger than the one with HI. We also observe that the distribution of the translocation velocity show a similar trend for polymers both with and without HI. The reason for this is since the translocation process in our simulations takes place under the forced translocation regime (the regime under **F**_elec_ >> *k*_B_T/*σ*_0_), the geometric shape of the polymer on the *cis* side remains almost the same as its initial configuration regardless of the level of the drag force. This means that there is not enough time for the polymer blob to relax from its initial shape while it is translocating.

**Figure 7:**
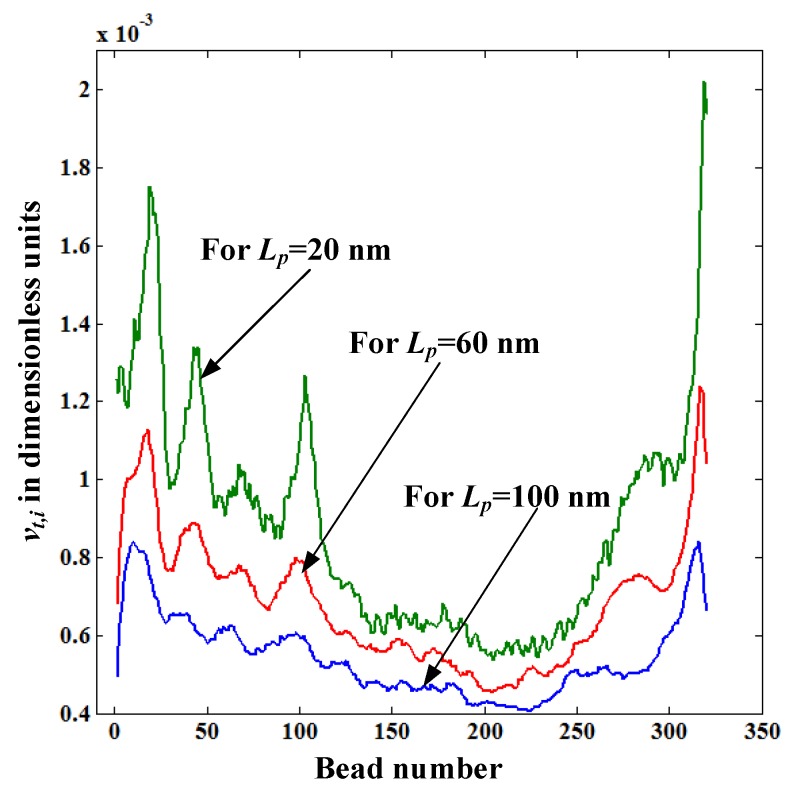
Variation of the translocation velocity of each bead *v_t_*_,*i*_ inside the nanopore for pore lengths *L_p_* = 20, 60, and 100 nm.

Figure 7 shows the distribution of translocation velocity of each bead *v_t_*_,*i*_ for pore lengths *L_p_* = 20, 60, and 100 nm. The value of *v_t_*_,*i*_ decreases with the pore length even though **F**_elec_ is the same for all *L_p_* values. The reason for this is that the drag force acting on the stretched portion of the polymer increases with the pore length as *l*_stretch_ increases with *L_p_* (see Figure 5). So, the main reason behind the increase of the translocation time with *L_p_* revealed in Figure 4 is due to the increased fluid resistance force caused by the increased length of the straightened portion of the polymer. In Figure 7, *v_t_*_,*i*_ values of the first and last few beads of the polymer are very low for *L_p_* = 60 and 100nm compared to those for *L_p_* = 20 nm. This is because at the beginning and the end of the translocation process a **F**_elec_ is lower for *L_p_* = 60, and 100 nm than for *L_p_* = 20 nm as the number of beads inside the pore for *L_p_* = 60, and 100 nm is reduced.

## 4. Conclusions

In this work, we developed a FORTRAN code to simulate the motion of a biopolymer (dsDNA) though a nanopore under an external electric field using different nanopore lengths. The polymer motion is simulated using LD equations and the fluid motion by LBE. The translocation time increases with the pore length even though the polymer receives the same electrophoretic force in all the pores regardless of their lengths. We found that the translocation velocity of each bead mainly depends on the length of the stretched portion of the polymer closest to the pore. We confirmed this using a theoretical formula. We also found that the increase of the translocation time with the pore length is attributed to the increased fluid drag force acting on the polymer due to the elongated stretching of the polymer within a longer pore.

## Supplementary Materials

Supplementary File 1
