# Supplementary material for: Effect of Nanopore Length on the Translocation Process of a Biopolymer: Numerical Study
Source: Materials (Basel). 2013 Sep 11;6(9):3989–4000. doi: 10.3390/ma6093989 (PMC5452664; doi:10.3390/ma6093989)
Supplement: Supplementary File 1 [file materials-06-03989-s002.pdf]

Supplementary Information

The force due to Lennard-Jones potential between the bead-bead and the bead-wall particle is computed by

$$\mathbf{F}_{\mathrm{LJ},i} = \begin{cases} 24\varepsilon \sum_{j=1\ \&\ j\neq i}^{N} 2\left(\frac{\sigma^{12}}{r_{i,j}^{13}}\right) - \left(\frac{\sigma^{6}}{r_{i,j}^{7}}\right)^{6} \hat{\mathbf{r}}_{i,j} & \text{if } r_{i,j} < r_{\mathrm{cut}} \\ 0 & \text{if } r_{i,j} \geq r_{\mathrm{cut}} \end{cases}, \tag{S1}$$

where σ and ε are the length and energy scales of the potential, respectively, and $r_{\mathrm{cut}} = 2^{1/6}\sigma$ is the cut-off distance which ensures that only repulsive interaction takes place when two beads are closer than r_{cut}. Further, $r_{i,j}$ denotes distance between the bead i and the bead j, and $\hat{\mathbf{r}}_{i,j}$ indicates the unit vector of the displacement vector $\mathbf{r}_{i,j}$ of the particle i referred to the particle j, *i.e.*, $\mathbf{r}_{i,j} = \mathbf{r}_i - \mathbf{r}_j$.

The bond stretching force due to the connectivity between the bead i and each of its adjacent beads, $i-1$ and $i+1$, is given by

$$\mathbf{F}_{\mathrm{bond},i} = -K_{\mathrm{bond}}(r_{i,i\text{-}1} - \sigma_0)\hat{\mathbf{r}}_{i,i-1} - K_{\mathrm{bond}}(r_{i,i+1} - \sigma_0)\hat{\mathbf{r}}_{i,i+1}, \tag{S2}$$

where K_{bond} is the bond-stretching force constant and σ_0 is the segment length. In our simulations, we have chosen $K_{\mathrm{bond}} = 1000\, k_{\mathrm{B}}T / \sigma_0^2$. Here k_{B} represents the Boltzmann constant and T is the temperature of the fluid.

The force due to bending interaction between the particle i and the two neighboring particles, $i-1$ and $i+1$, is calculated by

$$\mathbf{F}_{\mathrm{bend,i}} = -K_{\mathrm{bend}}(\phi - \phi_a)\left[\frac{1}{r_{i-1,i}} \frac{\mathbf{r}_{i-1,i} \times (\mathbf{r}_{i+1,i} \times \mathbf{r}_{i-1,i})}{\left|\mathbf{r}_{i-1,i} \times (\mathbf{r}_{i+1,i} \times \mathbf{r}_{i-1,i})\right|} + \frac{1}{r_{i+1,i}} \frac{\mathbf{r}_{i+1,i} \times (\mathbf{r}_{i-1,i} \times \mathbf{r}_{i+1,i})}{\left|\mathbf{r}_{i+1,i} \times (\mathbf{r}_{i-1,i} \times \mathbf{r}_{i+1,i})\right|}\right], \tag{S3}$$

where K_{bend} is the bending rigidity constant which restores the angle ϕ to an equilibrium angle $\phi_a = \pi$; here '$\times$' indicates the cross product. K_{bend} is related to the persistence length, P, of dsDNA and its value is $K_{\mathrm{bend}} = Pk_{\mathrm{B}}T / \sigma_0$.

The random force due to the thermal fluctuations in the fluid environment is taken as a Gaussian random variable with the mean and variance given by

$$\begin{aligned} &\left\langle \mathbf{F}_{\mathrm{ran},i}(t) \right\rangle = 0, \\ &\left\langle \mathbf{F}_{\mathrm{ran},i}(t)\mathbf{F}_{\mathrm{ran},j}(t') \right\rangle = 2\Gamma_{\mathrm{bare}} k_{\mathrm{B}} T \delta_{ij} \delta(t - t')' \end{aligned} \tag{S4}$$

where δ_{ij} and $\delta(t - t')$ are the Kronecker and Dirac delta functions, respectively. Here we replace Γ_{bare} with Γ when HI are neglected.

Table S1. Important simulation parameters.

Parameter	Value	Reference quantity	Dimensionless value
Equilibrium length of the segment, σ_0	$\sigma_0 = 10$ nm	σ_0	$\sigma_0^* = 1$
Lattice grid size, Δx	$\Delta x = 10$ nm	σ_0	$\Delta x^* = 1$
Density of water, ρ_o	$\rho_o = 1000$ kg/m^3	ρ_o	$\rho_o^* = 1$
Viscosity of water, μ	$\mu = 10^{-3}$ N-s/m^2	$\mu_{\text{ref}} = \dfrac{\mu}{\mu^*}$	$\mu^* = 7$
Temperature	$T = 300$ K	–	–
Energy, $\varepsilon = k_{\text{B}}T$	$k_{\text{B}}T = 4.14\times10^{-21}$ J	$k_{\text{B}}T$	$k_{\text{B}}T^* = \varepsilon^* = 1$
LB time step, Δt	$\Delta t = 6.9\times10^{-10}$ s	$\tau_{\text{ref}} = \dfrac{\mu_{\text{ref}}\sigma_0^3}{k_{\text{B}}T}$	$\Delta t^* = 0.02$ (LD time step, $\text{d}t^* = 0.01$)
Friction coefficient, Γ [From Equation (13) of Reference [21]]	$\Gamma = 4.61\times10^{-11}$ N-s/m	$\Gamma_{\text{ref}} = \mu_{\text{ref}}\sigma_0$	$\Gamma^* = 32$ ($\Gamma^*_{\text{bare}} = 40$)
Effective charge on each bead, q_i	$q_i = 1.42\times10^{-18}$ C	$q_{i,\text{ref}} = 1e$, where e is the proton charge	$q_i^* = 9$
Applied voltage, $\Delta\varphi$	$\Delta\varphi = 120$ mV	$\Delta\varphi_{\text{ref}} = k_{\text{B}}T / e$	$\Delta\varphi^* = 4.7$

© 2013 by the authors; licensee MDPI, Basel, Switzerland. This article is an open access article distributed under the terms and conditions of the Creative Commons Attribution license (http://creativecommons.org/licenses/by/3.0/).
